# Semaphorin 3A Increases in the Plasma of Women with Diminished Ovarian Reserve Who Respond Better to Controlled Ovarian Stimulation

**DOI:** 10.3390/life14030358

**Published:** 2024-03-08

**Authors:** Michela Palese, Gabriella Ferretti, Giuseppe Perruolo, Sara Serafini, Rossana Sirabella, Vincenzo Marrone, Martina De Rosa, Laura Sarno, Ida Strina, Carmela Matrone, Maurizio Guida

**Affiliations:** 1 Unit of Gynecology, Department of Neuroscience, School of Medicine, University of Naples “Federico II”, 80131 Naples, Italy; michela.palese@unina.it (M.P.); vincenzo.marrone@unina.it (V.M.); martina.derosa@unina.it (M.D.R.); laura.sarno@unina.it (L.S.); maurizio.guida@unina.it (M.G.); 2 Unit of Pharmacology, Department of Neuroscience, School of Medicine, University of Naples “Federico II”, 80131 Naples, Italy; gabriella.ferretti@unina.it (G.F.); sara.serafini@unina.it (S.S.); rossana.sirabella@unina.it (R.S.); 3 Department of Translational Medical Sciences, University of Naples “Federico II”, 80131 Naples, Italy; giuseppe.perruolo@unina.it; 4 Unit of Gynecology, Department of Hygiene and Public Health, School of Medicine, University of Naples “Federico II”, 80131 Naples, Italy; ida.strina@unina.it

**Keywords:** semaphorin 3A (SEMA3A), diminished ovarian reserve (DOR), in vitro fertilization (IVF), infertility, Gonadotropin-Releasing Hormone (GnRH)

## Abstract

Semaphorin 3A (SEMA3A) plays a crucial role in the development, differentiation, and plasticity of specific types of neurons that secrete Gonadotropin-Releasing Hormone (GnRH) and regulates the acquisition and maintenance of reproductive competence in humans and mice. Its insufficient expression has been linked to reproductive disorders in humans, which are characterized by reduced or failed sexual competence. Various mutations, polymorphisms, and alternatively spliced variants of SEMA3A have been associated with infertility. One of the common causes of infertility in women of reproductive age is diminished ovarian reserve (DOR), characterized by a reduced ovarian follicular pool. Despite its clinical significance, there are no universally accepted diagnostic criteria or therapeutic interventions for DOR. In this study, we analyzed the SEMA3A plasma levels in 77 women and investigated their potential role in influencing fertility in patients with DOR. The results revealed that the SEMA3A levels were significantly higher in patients with DOR than in healthy volunteers. Furthermore, the SEMA3A levels were increased in patients who underwent fertility treatment and had positive Beta-Human Chorionic Gonadotropin (βHCG) values (β+) after controlled ovarian stimulation (COS) compared to those who had negative βHCG values (β−). These findings may serve as the basis for future investigations into the diagnosis of infertility and emphasize new possibilities for the SEMA3A-related treatment of sexual hormonal dysfunction that leads to infertility.

## 1. Introduction

Ovarian reserve refers to the collection of follicles present in a woman’s ovaries at a particular point in her life. This reserve is naturally and irreversibly depleted over time because of normal ovarian aging (NOA). With advancing age, the quantity and quality of eggs decrease; however, in some cases, women experience a reduced ovarian reserve (DOR) earlier than usual, leading to premature infertility [1].

Currently, there is no universally accepted definition for DOR, as acknowledged by the American Society for Reproductive Medicine Practice Committee (2012). However, clinical practitioners typically diagnose DOR by assessing changes in ovarian reserve tests, such as low levels of anti-Müllerian hormone (AMH) and a low antral follicular count (AFC), elevated baseline follicle-stimulating hormone (FSH) levels (not exceeding menopausal levels), or, less frequently, clomiphene citrate test failure in women with regular menstrual cycles [1].

DOR may precede premature ovarian insufficiency (POI), a clinical syndrome that affects 1% of the female population and is characterized by the loss of ovarian activity before the age of 40 years. Unlike DOR, POI has well-defined diagnostic criteria, as outlined in the European Society of Human Reproduction and Embryology (ESHRE) guidelines. In particular, a diagnosis of POI is typically confirmed in women under the age of 40 years after a period of amenorrhea or oligomenorrhea lasting at least 4–6 months and according to a combination of elevated levels of gonadotropins, FSH > 25 IU/L, and decreased estradiol (E2) [2].

The etiology of this condition varies significantly. Although chromosomal and genetic defects; iatrogenic insults (such as surgery, radiation therapy, or chemotherapy) and autoimmune disorders or infections, have been frequently associated with POI, in most women, the causative factor for this syndrome remains unknown. These conditions are commonly referred to as idiopathic POI [2]. The same principle can be applied to patients with premature DOR; in most cases, the cause of the disease remains unclear.

Although they represent two conditions closely connected to each other, DOR and POI should be considered two different pathological entities that require different management, not only to satisfy reproductive desire but also to protect the general state of health. Both conditions expose women to poor reproductive prognosis, which is clearly worsened by the age at which they seek pregnancy and the severity of the condition itself. Reduced ovarian reserve determines a poor response to controlled ovarian stimulation (COS) in in vitro fertilization (IVF) techniques, a reduced number of oocytes retrieved at pick-up, and therefore, a low number of blastocysts. Therefore, IVF techniques with ovo-donation represent the most effective therapeutic strategy for women with poor follicular patterns, although patients are unlikely to accept this treatment, especially if they have never undergone homologous fertilization.

Therefore, the intricacy of the DOR condition necessitates the discovery of molecular signatures that predict the condition’s outcome, thereby facilitating the characterization of clinical profiles that are either responsive or unresponsive to therapy. Moreover, these signatures may aid in identifying individuals who will benefit from personalized treatment strategies.

In this regard, an increasing number of studies point to the family of semaphorins (SEMA) as potential candidates for the development of new diagnostic criteria for DOR, in association with those already used in routine clinical practice. SEMA is a family of proteins categorized into eight classes (SEMA 1–8) and is largely conserved across animal species [3]. The function of SEMA proteins has evolved to encompass more than just their initial identification as neuronal guidance cues. These proteins are now known to play crucial roles in the development and maintenance of various tissue types according to both short- and long-range interactions [4]. The diverse array of receptors, including plexins (PLXNs) and its coreceptors, such as neuropilins (NRPs), and various intracellular signaling components involved in SEMA signaling, contribute to their versatility. Examination of the SEMA function within neural and vascular systems offers valuable insights into the regulatory principles that influence the role of this protein family in physiological processes and diseases.

In particular, an increasing number of studies have emphasized the effects of class 3 SEMA (SEMA3) on the motility, survival, and axonal plasticity of neurons that secrete Gonadotropin-Releasing Hormone (GnRH) [5,6,7].

Four members of the SEMA3 family, including SEMA3A, SEMA3B, SEMA3C, and SEMA3F, have been found to be expressed in and around the developing olfactory/vomeronasal system [5]. In particular, SEMA3A regulates the migration of GnRH-secreting neurons from the nasal placode to the hypothalamus, which controls the pulsatile secretion of GnRH until adulthood [8]. NRPs and PLXNs are expressed in the olfactory system. Specifically, Nrp1 and 2 are expressed by the sensory neurons in the main and accessory olfactory epithelia (OE) of rodents and zebrafish, whereas PlexinA1 is highly expressed in vomeronasal organs (VNOs) and VNNs [8]. Notably, when the SEMA3 signal is interrupted or absent, the structure and function of the GnRH system is altered. For instance, in mice lacking Nrp2, abnormal accumulation of GnRH neurons in the nasal compartment occurs, potentially due to the defasciculation of the olfactory/vomeronasal axons and the subsequent failure of the neurons to migrate to their forebrain destinations [9,10]. This deficit in the GnRH neurons at their final location results in infertility [11].

Interestingly, mutations in *SEMA3A*, *SEMA3E*, *SEMA7A*, and their receptors (*PLXNA1*, *NRP1*, and *NRP2*) have been identified in patients with isolated GnRH deficiency [9,12,13,14,15,16] or Kallmann Syndrome (KS) [13,17]. Young et al. described eight different mutations in the heterozygous state of *SEMA3A* in 6% of patients with KS [13,17]. Additionally, they reported a large deletion in *SEMA3A* associated with KS in a heterozygous state in two siblings and their clinically affected fathers [9,13,17]. Furthermore, SEMA3A has been linked to female reproductive failure due to an autosomal dominant mutation that causes hypogonadotropic hypogonadism (HH) [18,19]. 

SEMA3A, SEMA6A, and SEMA6D play crucial roles in cell–cell communication, particularly in oocyte cumulus complex maturation [20]. Yan et al. demonstrated that the suppression of SEMA promotes preantral follicle atresia, which may have significant implications for follicular development [21]. This could be particularly relevant in the context of the response to gonadotropins for multiple follicular development, which is typically desired in hormonal stimulation treatments.

In addition, SEMA inhibition caused a significant decrease in estradiol, progesterone, and testosterone levels in vivo [22]. Transcriptome sequencing analysis revealed that SEMA4C caused alterations in the pathways related to ovarian steroidogenesis and the actin cytoskeleton [23]. Knockdown of SEMA4C using siRNA in mouse primary ovarian granulosa cells or thecal interstitial cells significantly impaired ovarian steroidogenesis and caused disorganization of the actin cytoskeleton [23].

Notably, previous research has shown that SEMA3A, which functions as a tumor suppressor, is frequently downregulated in various malignancies, including ovarian epithelial carcinomas [24,25]. As a result, decreased SEMA3A expression has been proposed as a prognostic factor for poor patient survival [25]. These findings underscore the clinical relevance of SEMA3A in ovarian cancer prognosis and highlight its potential as a biomarker and therapeutic target not only in mitigating tumor progression and metastasis but also in other ovarian-associated conditions, such as DOR.

The objective of this study was to investigate the levels of SEMA3A in the plasma of patients with DOR and to determine whether there were any differences in these levels between those who responded better to COS in the context of IVF techniques. 

## 2. Materials and Methods

### 2.1. Study Design and Patient Recruitment

This study was conducted at the “Infertility Couple Center” of the Department of Gynecology and Obstetrics, at the “Federico II” University (Naples, Italy). Before enrolment, all the participants signed a written consent form. All the procedures followed the guidelines outlined in the Declaration of Helsinki. The study protocol was approved by the local ethics committee of “Federico II” University of Naples (protocol no.79/2023). A total of 223 infertile women were screened. Among the screened population, 77 women aged 25–38 years were included in the study. A total of 42 of them presented with a history of infertility for at least one year and AMH ≤ 1.2 ng/mL and/or AFC ≤ 7, both assessed during the same menstrual cycle and in the early follicular phase (Table 1). AFC was determined using two-dimensional (2D) transvaginal ultrasonography according to the practical recommendations for the standardized use of AFC [26]. All the doctors the performing AFC assessments had formal training in ultrasonography and reproductive medicine and a minimum of three years of experience in the field. The scans were obtained using a 4–10 MHz endocavitary transducer (GE Healthcare, Milwaukee, WI, USA). The number and diameter of follicles were assessed for each ovary. To better investigate the severity of ovarian reserve depletion and the degree of ovarian dysfunction, hypothalamic–pituitary–ovarian (HPO) axis functionality was investigated by assessing the FSH, luteinizing hormone (LH), and E2 levels on days 2–4 of the menstrual cycle. Other pituitary trophins that might regulate ovarian function and response to COS, such as prolactin (PRL) and thyroid-stimulating hormone (TSH), were also measured [27].

Patients with a history of ovarian surgery, genital radiotherapy, or chemotherapy and suffering from diseases with a known impact on the ovarian reserve, such as endometriosis and autoimmune or genetic disorders (X-fragile pre-mutation, karyotype alterations), were not included in the study. All patients had normal weights (body mass index, BMI ≤ 30 kg/m^2^ and ≥18 kg/m^2^). Patients with psychiatric disorders, major depression, or mental retardation were also excluded.

All patients underwent an antagonist protocol except for one patient who underwent a natural cycle (Table 2). Gonadotropins were administered once daily on the second day of the menstrual cycle via the subcutaneous (s.c.) route. We determined the starting dose to be 150–300 IU based on age, AMH, AFC, BMI, and basal sex hormone levels. From day 6, 0.25 mg/day of the GnRH antagonist was administered s.c. once daily.

During the process of ovarian stimulation, we monitored the size of the follicles with transvaginal ultrasound and the levels of E2 and progesterone (P) and adjusted the dosage of stimulation every 2–3 days, if necessary. When at least one follicle reached a mean diameter of 18 mm, as well as 5000 or 10,000 IU of human chorionic gonadotropin (hCG), it was used for triggering. Oocyte retrieval was performed 34–36 h after transvaginal ultrasound guidance. All the patients underwent fresh embryo transfer. The embryos were transferred on day 3 after the oocyte retrieval; alternately, a single blastocyst was transferred on day 5 after the retrieval.

All the patients received luteal support using vaginal progesterone (200 mg/three times a day) for 14 days, starting on the day of oocyte retrieval and continuing until the day of β-hCG testing.

Fourteen patients underwent fresh embryo transfer (day 3 after oocyte retrieval), and seven underwent fresh blastocyst transfer (day 5 after oocyte retrieval). Pregnancy was achieved in eight patients. Two patients terminated their pregnancies because of miscarriage. Two of the patients had twin pregnancies (Table 2). Five cycles were cancelled: two due to oocyte recovery failure and three due to fertilization failure.

### 2.2. Plasma Collection

Blood samples (6 mL) using venipuncture were collected in BD Vacutainer^®^ CPT™ Mononuclear Cell Preparation Tubes (#BD 362753) containing sodium heparin. The blood was centrifuged (1800 rpm for 30 min) to separate the plasma, peripheral blood mononuclear cells (PBMCs), and platelets from the denser blood components. Approximately 5 mL of plasma was collected and stored at −80 °C until use.

### 2.3. Enzyme-Linked Immunosorbent Assay (ELISA)

The SEMA3A protein levels in the plasma of each patient were assessed using an ELISA kit (Cusabio, #CSB-E15913h, Houston, TX, USA) following a previously reported procedure [28]. The kit sensitivity was 0.156 ng/mL, according to the manufacturer’s instructions. The SEMA3A values for each patient were expressed as the ratio between the SEMA3A levels and the total plasma protein content, as determined using Bradford assay. Quantification was performed twice in triplicate and expressed as the mean ± standard error of the mean (mean ± SEM).

### 2.4. Biochemical Analysis

The blood levels of E2, PRG, PRL, LU, FSH, and TSH were analyzed using an ADVIA Centaur XPT Immunoassay System analyzer (Siemens, Saint Paul, MN, USA) based on competitive (E2) or direct (FSH, LH, TSH, and PRL) immunoassays. Quantification of the reaction was performed using Chemiluminescent Acridinium Ester technology. The AMH levels were analyzed using an ELISA (Beckman-Coulter II Gen, Inc., Webster, TX, USA).

### 2.5. Statistical Analysis

GraphPad Prism software version 10.2 for Mac (GraphPad Software, San Diego, CA, USA) was used for the statistical analysis. The nonparametric Spearman’s correlation test was used to correlate and analyze multiple variables in the study groups. Statistical significance was set at *p* value < 0.05. The unpaired *t*-test or Fisher’s test was used to analyze the clinical features reported in Table 1, following the suggestions of the GraphPad 10.2 software.

## 3. Results

### SEMA3A Increases in Patients with DOR

A total of 77 women were divided into two groups: 35 healthy women (HLT) and 42 women with DOR. The clinical profiles of the study participants are presented in Table 1. The ovarian reserve indicators most widely used in clinical practice include AMH levels and AFC. In addition, changes in the levels of FSH, LH, and E2 can support the prognostic evaluation of women with DOR [29,30]. The control group consisted of 35 healthy volunteers with no history of infertility or an ovarian reserve appropriate for reproductive age, as assessed using serum AMH assay and ultrasound evaluation of AFC performed during the early follicular phase (Table 1).

The two groups did not differ in terms of age or age at menarche. However, the DOR group had a higher BMI than the HLT group did. In addition, a larger number of patients with DOR had thyropathies, which are considered risk factors for DOR [31]. Notably, significant differences in the AFC, AMH, FSH, LH, PRL, E2, and TSH levels were found between the two groups (Table 1).

**Figure 1:**
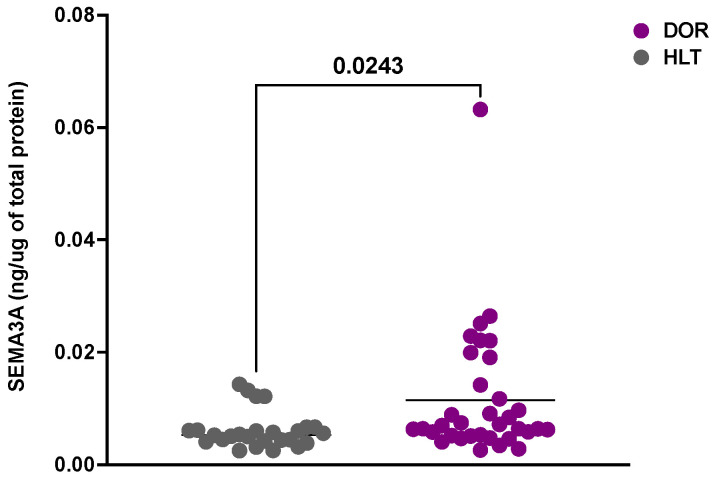
SEMA3A increases in patients with DOR. SEMA3A levels in HLT and patients with DOR. Significance was calculated using unpaired *t*-test and expressed as a *p* value < 0.05. Data below the sensitivity of the kit were excluded.

We assessed the SEMA3A levels in the HLT and DOR groups. Seven patients in the HLT group and four in the DOR group showed values below the kit’s sensitivity and were therefore removed from the analysis. We found that the SEMA3A levels were higher in patients with DOR than in HLT subjects (HLT 0.0061 ± 0.0006; DOR 0.011 ± 0.002) (Figure 1).

We next focused on 26 patients who underwent COS followed by IVF, 14 patients who underwent fresh embryo transfer (day 3 after oocyte retrieval), and 7 patients who underwent fresh blastocyst transfer (day 5 after oocyte retrieval) (see Section 2).

The 26 patients were divided in three groups, those who had negative (β−) or positive (β+) βHCG values after COS and those that did not complete the procedure (cancelled cycle, CC). The E2 values and the number of follicles at the trigger and mature oocytes in the three groups are shown in Table 3.

**Figure 2:**
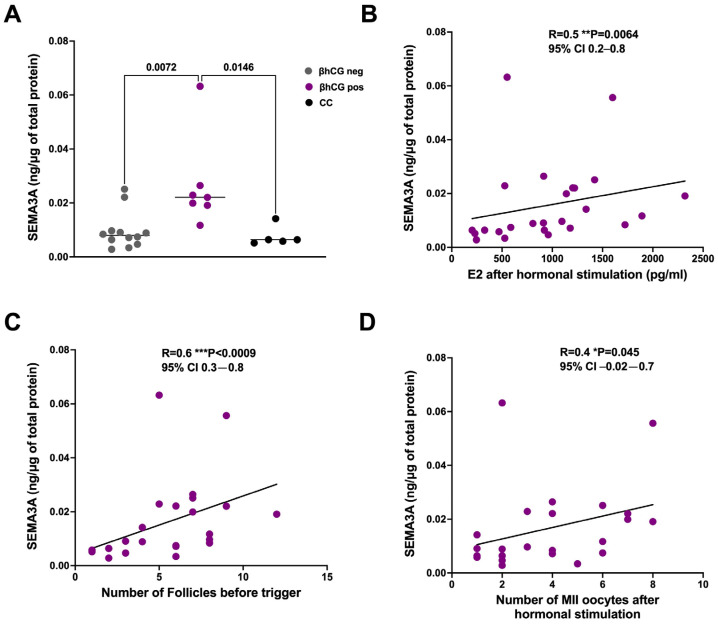
SEMA3A increases in patients with DOR who responded better to ovarian stimulation. (**A**) SEMA3A levels in patients with DOR who were responsive or unresponsive to fertilization procedures. βneg: negative-βhCG; βpos: positive-βhCG; CC: cancelled cycle. Significance was calculated using ordinary one-way ANOVA followed by Tukey’s multiple comparisons test and expressed as *p* value < 0.05. Data below the sensitivity of the ELISA kit were excluded from the analysis. Spearman’s correlation analysis between SEMA3A and E2 (**B**), the number of follicles before trigger (**C**), and mature oocytes (MII oocytes) (**D**) after hormonal stimulation in women with DOR. R = Spearman’s correlation coefficient; the confidence interval (CI) was set at 95%; *p* values are reported in the graph. * *p* < 0.05; ** *p* < 0.01; *** *p* < 0.001.

We compared the SEMA3A levels in these three groups and found that the SEMA3A expression was significantly higher in the β+ patients than in the β− or CC patients (Figure 2A and Table 3). In addition, the SEMA3A levels were positively correlated with the E2 levels (Figure 2B), number of follicles on the day of trigger, and number of mature oocytes in metaphase stage II (MII oocytes) retrieved at pick-up (Figure 2C,D).

## 4. Discussion

SEMA3A has recently attracted interest as a potential signature of defects or deficits in the reproductive system. This is mostly because mutations in its gene and receptors have been identified in patients with isolated gonadotropin deficiency and KS [12]. SEMA3A promotes the development of GnRH axons in the adult brain and plays a vital role in regulating central reproduction. Additionally, SEMA3A controls the secretory activity of gonadotropins in the GnRH neurons that are crucial to female fertility [5]. Of note, SEMA3A, which is secreted by the fenestrated endothelial cells of the hypothalamus–hypophyseal portal blood vessels, is believed to control the release of neuropeptides at critical moments in the ovarian cycle, such as during proestrus, when the preovulatory surge in GnRH occurs [6]. Interestingly, SEMA3A and SEMA3C expression is markedly increased in women with endometriosis [32]. SEMA3A was found in greater amounts in the glandular epithelial cells in peritoneal endometriosis and deep infiltrating endometriotic lesions of the uterosacral ligament [33]. This indicates that SEMA3A, as a nerve-repellent factor, may be responsible for the altered sympathetic innervation of endometriosis.

Notably, SEMA3A has also been implicated in conditions that are frequently associated with DOR, such as obesity. Genetic variations in SEMA3A have been reported to be associated with severe early onset obesity owing to disrupted SEMA3A signaling in pro-opiomelanocortin (POMC)-expressing anorexigenic neurons [34]. In support of this evidence, a lack of Nrp1 in GnRH neurons in mice leads to obesity, thus revealing the previously unrecognized role of SEMA3A and the GnRH system in maintaining energy homeostasis. Interestingly, the patients included in our study showed a BMI higher than that of the corresponding healthy controls, further underlining the potential crosstalk between fertility and SEMA3A.

Similarly, alterations in the thyroid gland function have been associated with infertility in women. High SEMA3A levels have been reported in patients with hypothyroidism compared to those in the euthyroid group [35]. In addition, proteins included in the SEMA3A downstream signal, such as CRMP1, 2, and 3, regulate processes related to thyroid function and are implicated in neonatal hypothyroidism [36].

The main finding of this short study is that the SEMA3A levels were increased in patients with DOR who responded better to COS, resulting in higher E2 levels and an increased number of follicles and MII oocytes. To the best of our knowledge, the potential of SEMA3A as a prognostic factor for improved outcomes in COS therapy has not been highlighted in the previous research, underscoring the necessity of further investigating this novel evidence. Although AMH levels and AFC are highly predictive and are typically utilized as first-line tests to counsel patients undergoing IVF procedures, their ability to determine the potential outcome of COS therapy is limited in terms of their prognostic value [36,37]. Therefore, it is important to integrate different biomarkers and conduct individualized evaluations to establish the predictive significance of different markers and to increase the effectiveness of treatment. It is reasonable to hypothesize that SEMA3A levels regulate the ovarian response to gonadotropins in women who undergo COS, most likely by influencing follicle development and oocyte maturation. This hypothesis appears to be consistent with other research that has linked changes in SEMA levels to various pregnancy-related pathologies [37,38]. In this regard, high SEMA3C levels have been found at the maternal–fetal–placental interface during the first trimester of gestation [39], and SEMA3F, SEMA3B, and NRP2 are overexpressed in the placenta of women with preeclampsia [37,40]. Interestingly, Xu et al. (2022) [33] revealed that the expression of SEMA3A was decreased in the decidua of patients who experienced unexplained spontaneous miscarriage. This finding suggests that SEMA3A may have a positive impact on embryo implantation during pregnancy and likely implies that SEMA3A has an impact on aspects of reproduction that involve fetal implantation and continuation of pregnancy. This is very important, especially if we consider that women with reduced ovarian reserve have poor responses to COS and poor pregnancy rates, even during IVF.

The possibility of distinguishing between patients with DOR based on specific characteristics or features might pave the way for personalized therapy and improve the outcomes of fertilization strategies.

## 5. Conclusions

Overall, this study sheds light on SEMA3A as a new player involved in the endocrine physiology of female reproduction and proposes SEMA3A as a novel diagnostic tool to identify a specific subset of patients with DOR who are likely to prove improved responsiveness to COS. The elevated levels of SEMA3A observed in DOR patients undergoing IVF may provide valuable insights into the potential for pregnancy and serve as a basis for individualized diagnostic and therapeutic strategies.

Indeed, the small sample size precluded our ability to draw sweeping conclusions, yet these findings should definitely spur further investigation into the potential utility of SEMA3A as a biomarker in identifying patients with DOR who may benefit from COS. To this end, it is essential to note that the cost-effectiveness of SEMA3A dosage is extremely good, making it feasible to incorporate an ELISA test on the plasma or sera of patients as part of the standard diagnostic procedures that always come before any therapeutic intervention. Of note, the heterogeneous nature of the patient population poses a challenge in determining whether patients with different SEMA3A levels would benefit most from COS therapy; however, it highlights the need to develop tailored diagnostic methods, which could ultimately result in personalized therapeutic approaches to individual patients.

## Figures and Tables

**Table 1:** Descriptive clinical profiles of the subjects enrolled in the study. HLT, healthy subjects; DOR, patients with diminished ovarian reserve. Data are expressed as mean ± standard error of the mean (MV ± SEM) or number upon total (N/Total). BMI, body mass index; TSH, thyroid-stimulating hormone; E2, estradiol hormone; FSH, follicle-stimulating hormone; LH, luteinizing hormone; PRL, prolactin; AFC, antral follicle count; AMH, anti-Müllerian hormone; ND, non-determined; NS, non-significant. Statistical analyses were performed using an unpaired *t*-test or Fisher’s exact test, following the suggestions of GraphPad 10.2 software. Significant *p* values are reported in the table. * *p* < 0.05; ** *p* < 0.01; **** *p* < 0.0001.

	HLT (*n* = 35)	DOR (*n* = 42)	*p* Value
**Clinical features (MV ± SEM)**			
Age (years)	32.17 ± 0.80	33.5 ± 0.51	NS
BMI (kg/m^2^)	22.86 ± 0.58	26.71 ± 1.00	** 0.0022
Age at Menarche (years)	11.57 ± 0.20	11.76 ± 0.20	NS
**Comorbidities (N/Total)**			Fisher’s Test
Thyropathy	3/35	12/42	* 0.0417
Hypothyroidism	2/35	7/42	
Hashimoto’s Thyroiditis	1/35	5/72	
Vitamin D Deficit	1/35	1/42	NS
Polycystic Ovary Syn- drome (PCOS)	2/35	0/42	NS
Fibroadenomas	0/35	3/42	NS
Mild Hyperprolactinemia	1/35	2/42	NS
Smoking	6/35	14/42	NS
**Drugs (N/Total)**			
Antihypertensives	1/35	0/42	NS
Thyroid Regulators	4/35	12/42	NS
Contraceptives	0/35	0/42	NS
Prolactin Inhibitors	0/35	1/42	NS
Anticonvulsants	0/35	0/42	NS
**Hormonal panel (MV ± SEM)**			Unpaired *T* test
E2 (pg/mL)	69.41 ± 9.80	48.50 ± 7.32	**** <0.0001
PRL (ng/mL)	3.77 ± 0.34	18.05 ± 2.45	* 0.0186
LH (mIU/mL)	2.73 ± 0.36	7.13 ± 1.66	**** <0.0001
FSH (mIU/mL)	2.94 ± 0.22	10.39 ± 0.97	**** <0.0001
TSH (µIU/mL)	0.59 ± 0.04	2.019 ± 0.18	**** <0.0001
**DOR markers (MV ± SEM)**			
AFC	20.00 ± 1.89	6.57 ± 0.48	**** <0.0001
AMH (ng/mL)	3.50 ± 0.45	0.85 ± 0.11	**** <0.0001
Sterility (years)	ND	3.86 ± 0.40	ND
**Sterility Cause (N/Total)**			
DOR	ND	22/42	ND
DOR + Tube Dysfunctions	ND	5/42	ND
DOR + Male Sterility	ND	15/42	ND
Primary Sterility	ND	27/42	ND
Secondary (Previous Pregnancies)	ND	15/42	ND

**Table 2:** IVF procedure in patients with DOR.

Patients with DOR Undergoing IVF Procedure (*n* = 26)	
**IVF procedure**	
Controlled ovarian stimulation (COS)	25
Natural cycle	1
**Kind of gonadotrophins**	
Recombinant FSH (rFSH)	8
Postmenopausal human gonadotropin, HMG	12
HMG + rFSH	5
**Cancelled cycle**	5
No oocytes retrieved	2
No fertilized embryos	3
**Fresh embryo transfer**	21
Embryo (day 3)	14
Blastocyst (day 5)	7

**Table 3:** SEMA3A, E2, number of follicles, and number of MII oocytes in the negative-βhCG-, positive-βhCG, and cancelled cycle groups. βneg: -negative-βhCG; βpos: positive-βhCG; CC: cancelled cycle; NS, non-significant. To evaluate statistical differences, ordinary one-way ANOVA followed by Tukey’s multiple comparison test was used. Statistical significance was expressed as *p* values. * *p* < 0.05; ** *p* < 0.01; *** *p* < 0.001.

	βneg (*n* = 13)	βpos (*n* = 8)	CC (*n* = 5)	*p* Value
SEMA3A levels (ng/μg of total proteins)	** 0.0096 ± 0.007	0.026 ± 0.016	* 0.0075 ± 0.0016	** 0.0072 vs. β+ * 0.0146 vs. β+
Estradiol at trigger (pg/mL)	* 968.15 ± 128	*** 1267.87 ± 223	632 ± 218	* 0.044 vs. CC *** 0.0002 vs. CC
Num. of follicles at trigger	5.38 ± 4.24	6.87 ± 2.82	2 ± 2.12	NS
Num. of MII oocytes	3.76 ± 0.70	4.75 ± 1.41	0.6 ± 0.70	NS

## Data Availability

Additional information about this study is available from the lead contact upon request.
